# Peutz-Jeghers Syndrome: A Comprehensive Review of Genetics, Clinical Features, and Management Approaches

**DOI:** 10.7759/cureus.58887

**Published:** 2024-04-24

**Authors:** Rohan L Amru, Archana Dhok

**Affiliations:** 1 Biochemistry, Jawaharlal Nehru Medical College, Datta Meghe Institute of Higher Education and Research, Wardha, IND

**Keywords:** chromosome 19p13.3, germline mutation, stk11 gene, mucocutaneous pigmentation, intestinal hamartomatous polyps

## Abstract

A relatively rare inherited condition known as Peutz-Jeghers syndrome (PJS) causes mucocutaneous pigmentation and gastrointestinal hamartomatous polyps. These polyps are non-cancerous, but the presence of PJS significantly increases the chances of developing various types of cancers, such as colorectal, pancreatic, gastric, and breast cancer. The purpose of this review article is to give an abbreviated summary of what is currently known about this syndrome, covering its clinical symptoms, pathophysiology, genetics, and management. PJS also raises the risk of getting many malignancies, especially gastrointestinal and pelvic cancers. Symptoms of the gastrointestinal tract brought on by hamartomatous polyps are frequent and include stool blockage, bleeding, and stomach pain. The pigmentation commonly appears as prominent bluish-black macules and frequently affects the skin and mucous membranes. Small macules and large regions of lentiginous pigmentation are both possible. Numerous areas, including the perioral area, buccal mucosa, fingers, and lips, exhibit pigmentation. Bowel obstruction and intussusception risk can be decreased by early identification and routine surveillance of gastrointestinal polyps. The gene serine/threonine kinase 11 (STK11) controls several biological functions, including cell polarity, growth, and proliferation. Genetic counseling is recommended for the affected individuals and their families. This can help assess the risk of passing on the condition to future generations and provide information about available reproductive options. Regular surveillance is crucial for managing the syndrome and reducing the risk of cancer development. Other syndromes and extra-gastrointestinal characteristics, such as somatic tumor polyps outside the gastrointestinal tract, are also linked to this syndrome.

## Introduction and background

Peutz-Jeghers syndrome (PJS) is a rare genetic disorder characterized by the development of gastrointestinal polyps, mucocutaneous pigmentation, and an increased risk of various types of cancer [1]. It was first described by Dr. Jan Peutz in 1921, who reported a family with multiple affected individuals presenting with intestinal polyps and associated pigmentation. In 1949, Dr. Harold Jeghers expanded upon Peutz’s findings and recognized the syndrome as a distinct clinical entity [2]. The defining feature of PJS is the presence of hamartomatous polyps in the gastrointestinal tract. These polyps typically appear as smooth, lobulated growths and are mostly found in the small intestine but can also occur in the stomach, colon, and other parts of the digestive system [1]. Another characteristic feature is the presence of mucocutaneous pigmentation, which appears as dark blue or brown macules on the lips, buccal mucosa, perioral region, genitalia, and fingertips [3]. Over the years, advancements in genetic research have shed light on the etiology of PJS. Researchers identified germline mutations in the serine/threonine kinase 11 (STK11) gene in individuals with PJS [4]. The STK11 gene, located on chromosome 19p13.3, encodes a serine/threonine kinase involved in cell growth regulation and tumor suppression [5]. Loss-of-function mutations in the STK11 gene lead to dysregulated cell growth and division, contributing to the development of polyps and increasing the risk of cancer in individuals with PJS [6]. Clinical and molecular research on PJS has significantly advanced the understanding of its pathogenesis, clinical manifestations, and management strategies. However, many aspects of the syndrome remain to be explored, including the role of modifier genes, molecular pathways involved in tumorigenesis, and the development of novel therapeutic approaches. Through ongoing research efforts, a comprehensive understanding of PJS will continue to evolve, potentially leading to improved diagnostic techniques, surveillance guidelines, and targeted treatments.

Overview of the genetic basis and inheritance pattern 

PJS is primarily caused by germline mutations in the STK11 gene, also known as LKB1 [5]. The STK11 gene is located on chromosome 19p13.3 and encodes a serine/threonine kinase that plays a crucial role in cell growth regulation and tumor suppression [6]. Loss-of-function mutations in STK11 disrupt the normal functioning of the gene, causing PJS-related traits, including gastrointestinal polyps as well as a higher risk of cancer to emerge [7]. PJS is inherited autosomal-dominantly, which means that each child of a person with the disorder has a 50% probability of inheriting the mutant gene. However, approximately 30% of cases are attributed to spontaneous mutations [8]. Many affected individuals have an affected parent, while in other cases, the condition arises from de novo mutations. It is important to note that individuals with PJS exhibit variable expressivity, meaning that the phenotypic manifestations can vary significantly even among family members carrying the same mutation [9]. This variability is attributed to factors such as genetic modifiers, environmental influences, and somatic mutations occurring during an individual’s lifetime [10]. A few percent of PJS cases have been shown to exhibit further genetic changes along with STK11 mutations. Along with unique mutations in other genes implicated in cell signaling such TGFBR1, BMPR1A, and ENG [6,11], these include significant genomic deletions covering the STK11 region. A majority of the PJS cases, however, are caused by STK11 germline mutations, and other genetic changes account for only a small portion of this disorder. 

## Review

Search methodology 

**Figure 1:**
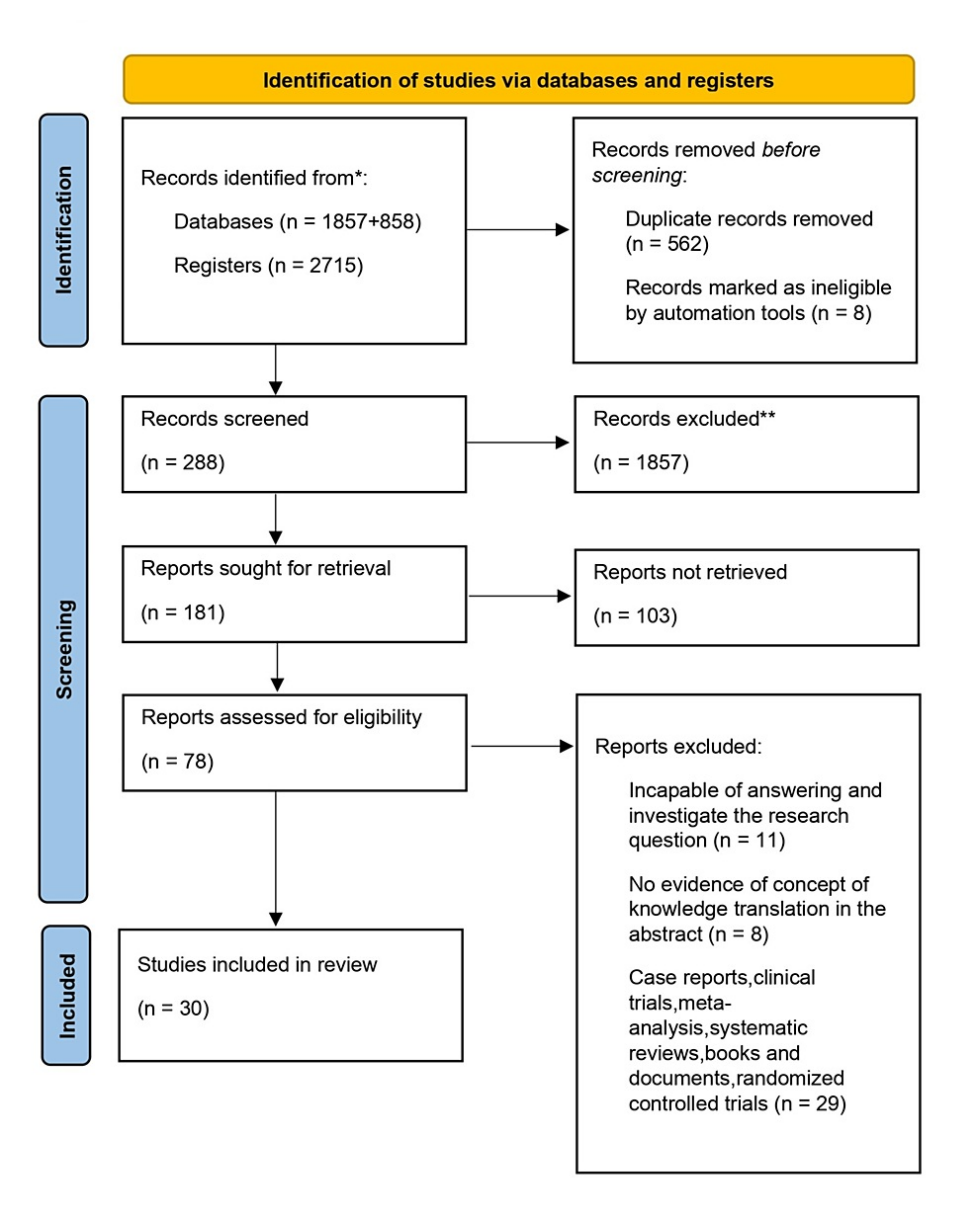
The selection process for articles used in this study Adopted from the Preferred Reporting Items for Systemic Reviews and Meta-Analyses (PRISMA) guidelines

We reviewed Scopus, PubMed, and Web of Science databases for articles on PJS pertaining to its management and health outcomes. We utilized as much pertinent research as we could find using the medical subject heading phrases "Peutz Jeghers syndrome", "tumor biomarkers", "chromosome 19p13.3", and "STK11/LKB1 gene" as well as various word combinations. A literature search was done to find case-control studies and meta-analyses in order to find any other records from other sources that might be relevant, as mentioned in Figure 1.

Clinical manifestations 

Gastrointestinal Polyps: Locations, Morphology, and Incidence 

This disorder elevates the probability of digestive system polyps development, which is a defining characteristic of PJS. These hamartomatous polyps can be seen throughout the digestive tract, with a preference for the small intestine. However, these polyps are additionally found within the stomach, colon, and other parts of the gastrointestinal tract. With regard to PJS, the small intestine accounts for about 75% of cases and is the location where polyps most frequently form [12]. The proximal jejunum, distal ileum, and duodenum are the areas of the small intestine where polyps are most common. With roughly 25% of PJS patients having gastric polyps, the stomach is the second most often affected site [13]. 

Mucocutaneous Pigmentation and Its Characteristics 

Gastrointestinal polyposis and unusual cutaneous features are hallmarks of the autosomal-dominant condition in PJS, which also has mucocutaneous pigmentation as a distinguishing feature. Its distinguishing characteristic is a smooth muscle core that extends into the polyp from the muscularis mucosae [14]. The skin and mucous membranes are frequently affected by pigmentation, which typically manifests as distinct bluish-black macules [12]. The perioral region, buccal mucosa, fingers, and lips are the most often impacted locations. Generally present at birth or developing in early childhood, the mucocutaneous pigmentation associated with PJS generally lasts into adulthood [15]. Described as freckle-like or lentiginous, the pigmented lesions have a distinctive appearance. Small macules and big patches can both be found among them, and their borders may be erratic. When PJS is suspected, mucocutaneous pigmentation is a crucial diagnostic indicator. Its existence can trigger additional investigation, genetic testing, and appropriate care, especially when linked to a favorable family history. Intussusception and bowel obstruction risk are reduced by early detection of mucocutaneous pigmentation and regular surveillance for gastrointestinal polyps. The results from bisulfite polymerase chain reaction (PCR), followed by Sanger sequencing, showed that PJS polyps had much higher levels of methylation of the LKB1 promoter than did normal colon samples [16]. If hamartomatous polyps are not visible on the first endoscopy, the diagnosis of PJS shouldn’t be ruled out because of the possibility that certain individuals could potentially be malnourished to some extent [17]. Endoscopies are more frequently performed to look for polyps that could cause intussusception or obstruction in the future than to look for tumors, but monitoring for the many cancers to which these people are predisposed is a crucial aspect of their potential therapy [18]. 

Extra-Gastrointestinal Features 

While PJS is primarily characterized by the presence of gastrointestinal polyposis, it is also associated with various extra-gastrointestinal features and other syndromes. Mucocutaneous pigmentation was previously noted as a distinguishing characteristic of PJS and is essential to the identification and diagnosis of the condition. PJS can manifest as pigmented macules or patches in other areas in addition to the typical spread-out pigmented lesions on the lips, buccal mucosa, and perioral region. The conjunctiva, perianal region, palms, soles, and vaginal region are other locations where macules or pigmentation can occur. PJS has been linked to the emergence of several somatic tumor polyps away from the gastrointestinal tract. Tumors of the thyroid, uterus, cervix, breast, small intestine, and pancreas are a few examples [15]. Because other illnesses like lung cancer can mimic this syndrome and even exhibit similar features on comparable scans, diagnosing lung cancer can be difficult. Particularly for those who have PJS, identifying the cancerous lung is crucial. Some lung growths in PJS patients may initially appear to be cancerous because both lung cancer and PJS influence comparable developmental pathways in the respiratory system. It's crucial to remember, though, that the instances included in this particular study were PJS-related. It draws attention to the necessity for additional studies on lung cancer mimickers connected to PJS in particular. This highlights how crucial it is to figure out any other explanation when a patient with PJS exhibits symptoms similar to lung cancer [19]. 

Cancer risk and epidemiology 

Gastrointestinal Cancers 

By the age of 70, PJS patients are at an increased risk of having colorectal cancer by about 39% [15]. With cumulative chances ranging from 13% to 36%, PJS is linked to a markedly higher probability of small bowel cancer [7]. At the age of 70, people with PJS are at an increased risk of developing stomach cancer by about 29% [15]. Recent data suggests that the total neoplastic transformation from PJS is not an unusual event despite the intestinal hamartomatous polyps having a lower incidence of malignant alteration in the gastrointestinal tract compared to adenomatous polyps [20]. When polyps grow repeatedly, they may produce life-threatening consequences like intestinal obstruction, intussusception, gastrointestinal bleeding, and cancerization, which have major clinical manifestations [21]. 

Non-Gastrointestinal Cancers 

PJS has been linked to a higher chance of developing pancreatic cancer, having an estimated 11% cumulative risk by age 55 [9]. From a young age, women are urged to get regular gynecological and breast screenings done [22]. Females with PJS are more likely to develop breast cancer than the general population, with the associated risks varying between 12% and 18% [23]. Although the reported incidence rates vary, PJS has also been linked to an increased risk of lung, liver, and various extra-intestinal malignancies [24]. PJS gene carriers are more likely to develop cancer both inside and outside the gastrointestinal tract in addition to the gastrointestinal issues brought on by polyps. Based on studies, PJS is linked to both gastrointestinal and non-gastrointestinal tumors [25]. 

Epidemiology 

PJS is autosomal-dominantly inherited and is estimated to affect one in 50,000 to one in 200,000 people [8]. PJS often manifests in early adolescence or in childhood, with a gender-neutral distribution [6]. PJS spans a wide range of ethnicities across the globe. Research is ongoing to determine if specific ethnicities have a higher prevalence.

Diagnostic criteria and genetic testing 

Clinical Criteria for Diagnosing Peutz-Jeghers Syndrome

PJS is characterized by the presence of one or more gastrointestinal polyps that have been histologically proved to be hamartomatous. Any region of the gastrointestinal tract can develop polyps, which can cause obstructive or bleeding symptoms. Multiple pigmented macules, which typically first emerge in childhood, are frequently the syndrome’s primary symptom. PJS often runs in families, and those who are affected exhibit an autosomal-dominant manner of inheritance. De novo mutations, on the other hand, can happen, resulting in singular cases without a family history. 

Genetic Testing Methods and Approaches

Identification of the underlying genetic etiology and confirmation of the PJS diagnosis depend heavily on genetic testing. The STK11/LKB1 gene, found on chromosome 19p13.3, is the most frequently implicated causal gene for PJS. The STK11 gene’s DNA sequence is the main tool for locating pathogenic mutations that cause PJS. Point mutations, minor insertions and deletions, and significant duplications and deletions can all be detected using sequencing techniques [5]. Genetic assistance is advised for people who have been suspected of having PJS or who have been handed the diagnosis in order to go over the advantages, restrictions, and consequences of genetic testing. It entails a thorough investigation of the family history, determining the possibility of transmission, and disclosing details regarding the pattern of inheritance and related cancer risks. Combining wireless capsule endoscopy, magnetic resonance enterography, and device-assisted enteroscopy is an effective way to reduce the number of polyps in the body, which lowers the risk of bleeding and intussusception [26]. 

Surveillance and management guidelines 

Upper Gastrointestinal Tract

The purpose of routine endoscopic screening for people with PJS is to ensure early detection and management of complications. Esophagogastroduodenoscopy (EGD) should be performed yearly or biannually beginning when a patient is younger or whenever digestive complaints arise. The upper gastrointestinal system can be seen during this operation, and polyps can be seen. Not every polyp needs to be removed in order to reduce the possibility of further complications. Endoscopic ultrasonography (EUS) can be utilized to measure polyp depth and direct treatment choices [27]. Capsule endoscopy has become an innovative approach that may be utilized for small intestinal examination, particularly when standard endoscopy and radiography are unable to offer a clear vision [28].

Colorectal Surveillance

Beginning at puberty or around age 15, or earlier, if an early-onset colorectal cancer is noted, annual colonoscopies should be performed. A colonoscopy should be done right away if there is gigantism and fast polyp development [29]. 

Cancer threat and management 

All the cases of PJS can’t be confirmed by mutations in the STK11/LKB1 gene. More investigation is required to find novel genetic variants connected to the condition [14]. Whole-genome sequencing research and next-generation sequencing methods offer the potential to identify new genetic changes [30]. An LKB1 mouse deletion model has generated a condition with phenotypical similarities. Numerous PJS kindreds lacking LKB1 mutations have been reported, pointing to other PJS loci. Understanding the functional effects of the discovered genetic variations and their relationship to illness severity and therapeutic results requires more research. Research should concentrate on improving the cancer risk estimation models that can more accurately forecast each person’s likelihood of contracting a particular malignancy linked to PJS [31]. New genetic markers, biomarkers, and clinical factors may enhance the precision and therapeutic usefulness of risk prediction models. Non-invasive screening procedures, such as imaging modalities like ultrasound, or a non-invasive and relatively inexpensive imaging modalities can be used to detect masses or lesions in the gastrointestinal tract and other organs, Computed tomographic (CT) scan is more detailed imaging modality that uses X-rays to create cross-sectional images of the body. CT scans are often used with contrast dye, which helps to highlight tumors and other abnormalities, can increase surveillance precision, lessen patient burdens, and help in the early diagnosis of cancer in individuals suffering from PJS [32]. A summary of all the articles included in this review is listed in Table 1.

**Table 1:** Summary of the articles included in the review

Authors	Year	Country	Findings
Richter [1]	2016	USA	Esophageal dilatation works well for lasting relief from eosinophilic esophagitis.
Jeghers et al. [2]	1949	UK	Identification of intestinal polys and associated pigmentation.
McGarrity et al. [3]	2021	USA	The presence of dark blue macules in various regions is a characteristic feature of hamartomatous polyps.
Mehenni et al. [4]	1997	Switzerland	Etiological basis of gene mutations.
Hemminki et al. [5]	1998	Italy	Germline mutations in gene on chromosome 19p.
Resta et al. [6]	2013	Finland	Gene mutations cause uncontrolled cell growth.
Aretz et al. [7]	2005	Germany	STK11 germline mutations and increase in the risk for cancer.
Boardman et al. [8]	1998	USA	Spontaneous mutations along with age onset.
Beggs et al. [9]	2010	UK	Genetic phenotypic manifestations among family members.
Lim et al. [10]	2004	UK	Factors like genetic modification, environmental influences, and somatic mutations can continue during life.
Matera et al. [11]	2020	Italy	Genetic mutations linked with cell signaling.
Maglinte et al. [12]	2008	USA	Small intestine has a higher risk for polyps development.
Simpson et al. [13]	2013	UK	Sites at the risk of polyps development.
Buck et al. [14]	1992	USA	Smooth muscle core extends into polyps, is a distinct characteristic.
Hearle et al. [15]	2006	UK	Early age onset of developing mucocutaneous pigmentation.
Li et al. [16]	2020	China	Methylation is linked to promoter and malignant tumors.
Ortegón-Gallareta et al. [17]	2022	Mexico	Nutritional assessment in diagnosing the patients.
Kitagawa et al. [20]	1995	USA	Monitoring tumors is a crucial aspect of therapy.
Korsse et al. [18]	2013	Netherlands	Intestinal hamartomatous polys are less malignant than adenomatous polys.
O’Riordan et al. [21]	2009	Ireland	Life-threatening consequences due to polyps growth.
Xu et al. [22]	2023	China	Regular gynecological and breast screening can help in early detection in females.
Giardiello et al. [23]	1987	USA	Affected females are more prone for developing cancer.
Gruber et al. [24]	1998	USA	Affected gene causes malignancies in various extra-intestinal areas.
Westerman et al. [25]	1999	Netherlands	Gene carriers can cause polyps development in different body locations.
Brosens et al. [27]	2007	Czech Republic	Combining various imaging techniques can reduce the number of polyps in the body.
Tacheci et al. [26]	2021	USA	Enterography and endoscopy combined can reduce the polyps risk.
van Lier et al. [28]	2010	Netherlands	Endoscopic procedure should be utilized for small intestinal examination.
McGarrity et al. [29]	2006	USA	Annual colonoscopies can detect early-onset colorectal cancers.
Syngal et al. [30]	2015	USA	To identify genetic changes, whole-genome sequencing can be helpful.
Skrzypczak et al. [31]	2010	Poland	To create colon tumor markers, oncogenic signaling should be identified.
Neacşu et al. [19]	2021	Romania	Association of lung mimickers in the disease with lung cancer.
Şerboiu et al. [32]	2023	Romania	Imaging modalities used in the detection of tumors.

Limitations of the present study 

Despite significant advancements, not enough is understood about PJS. A large-scale study recruiting many patients is difficult due to the disease's rarity, which limits the generalizability of the research findings. Furthermore, determining common ailments within PJS is complicated by its genetic variability. Targeted therapy development is further impeded by the current inadequate understanding of the role of PJS-associated genes. Lastly, other important factors, like the syndrome's association with several cancers, are obscured by the concentration of research on intestinal polyps. These limitations highlight the need for additional studies to enhance PJS management overall along with diagnosis and therapy. 

## Conclusions

PJS is a severe genomic illness defined as the growth of polyps in the gastrointestinal tract and an increased severity of several cancers. The etiology and genetics of PJS, its clinical manifestations and diagnostic standards, as well as management and surveillance suggestions for those affected by PJS, are the main topics covered in this review article. Overall, this review highlights the value of early detection and ongoing monitoring for those with PJS. We can reduce the chance of cancer development and enhance the patient's overall prognosis by following proper management measures.
